# Use of a Lateral Extended Nasal Island Flap for Nasal Reconstruction

**DOI:** 10.1001/jamafacial.2019.0423

**Published:** 2019-07-03

**Authors:** Simon J. Madorsky, An Ta

**Affiliations:** 1 Skin Cancer and Reconstructive Surgery Center, Newport Beach, California

## Abstract

**Importance:**

Small and medium nasal defects of the nose have been treated mostly with local skin flaps and skin grafts producing variable aesthetic outcomes. The lateral extended nasal island (LENI) myocutaneous flap provides a reliable single-stage reconstruction for small to medium nasal defects, including those in the nasal tip, with excellent cosmetic results.

**Objective:**

To describe and define a new version of the lateral nasal island flap for small to medium nasal defects.

**Design, Setting, and Participants:**

This retrospective case series included all patients who had undergone reconstruction with the lateral nasal island flaps and LENI flaps from October 2009 to July 2018 with 97 consecutive cases studied. Indications for surgery were mostly skin cancer defects. The analysis was performed from January 1, 2018, through December 15, 2018.

**Main Outcomes and Measures:**

The location and the size of the defects were defined. Number of stages required to achieve functional and aesthetic goals was reported. Appearance rating after the first stage was assessed.

**Results:**

The case series included 82 patients (mean age, 64 years [range, 31-90 years]; 36 [43%] women and 46 [57%] men). These 82 patients’ flaps qualified as the LENI flap. Of these, 54 (65%) were used for nasal tip reconstruction. The flap advancing distance ranged from 0.7 cm to 1.8 cm (mean advancing distance, 1.2 cm). Of 67 patients with available postoperative photographic documentation, 44 had mild or undetectable signs of reconstruction, 21 had visible scars, and 3 had obvious deformities. A single-stage procedure was performed in 66 cases, 2 stages in 15 cases, and 3 stages in 2 cases.

**Conclusions and Relevance:**

The LENI myocutaneous flap is a reliable technique for reconstruction of nasal defects up to 1.8 cm. The extended dissection and release of the flap at piriform aperture achieves the predictable outcome and separates it from the previously described lateral nasal island flaps.

**Level of Evidence:**

4.

## Key Points

**Question:**

What is the application of the lateral extended nasal island (LENI) flap and its cosmetic outcome in nasal reconstruction?

**Findings:**

This retrospective case series study of 82 patients who underwent the LENI flap procedure found that the LENI flap is a reliable single-stage reconstruction for various nasal defects including the nasal tip, bridging distances up to 1.8 cm.

**Meaning:**

The lateral extended nasal island flap is an effective and predictable single-stage reconstructive technique for medium-size nasal tip defects.

## Teaser

This case series examines the application of a novel lateral extended nasal island flap and its cosmetic outcome in nasal reconstruction of cosmetic defects.

## Introduction

Nasal reconstruction of cutaneous defects traditionally relies on methods such as linear closure, skin grafts, bilobe and other local flaps, nasolabial island flaps, and forehead flaps. Most of these flaps require excision of normal skin, wasting normal tissue. Some may cause distortion of the nasal shape, and some require multiple stages to achieve adequate results. Island flaps and the subset of lateral nasal island flaps have been described but have not reached popularity of the other techniques. Other names used to describe them includes V-Y advancement, nasalis myocutaneous island flap, alar island flap, and subcutaneous sliding flap.

Island flaps have been described in the literature as early as the nineteenth century.^1^ Called subcutaneous pedicle flaps and island flaps, they have been used throughout the face. Although Esser^1^ is often cited and credited with the earliest island flaps, Barron and Emmett^2^ reviewed the literature and identified authors and publications who described the technique as subcutaneous pedicle flaps in the 1800s. Zook et al^3^ in 1980 presented the island flap technique as V-Y advancement flap with photographic documentation of its use throughout the face.

Nasal island flaps were specifically described first in 1983 by Rybka,^4^ who set the stage with the description of the nasalis myocutaneous sliding flap performed in 47 patients. The blood supply was based on the aponeurosis of the nasalis muscle. He described a release of the deep nasalis attachments and cephalad muscle strands achieving a 1.25-cm defect closure primarily in the lateral nasal tip defects.^4^ Staahl^5^ reaffirmed the nasalis myocutaneous flap for supratip defects with incisions following the natural alar groove. He presented a handful of patient data with defects of 1.5 to 2 cm.^5^ Wee et al^6^ presented 19 nasalis myocutaneous flaps advancing 1.2 to 1.5 cm in the lateral nasal tip, ala, and nasal sidewall. They discuss the mobilization of the flap with strands of nasalis muscles bluntly dissected.^6^ In 2017, La Padula et al^7^ modified the nasalis flap by basing it on the superior muscle attachment, which they claimed allowed for a greater mobility of the flap.

Despite their long history, few island flaps have reached popularity this century, especially in nasal reconstruction. The heretofore described island flaps have poorly defined indications, limited applications, and vague technique descriptions. Their use is often a curiosity rather than a defined technique for a specific indication.

Over the past decade, the senior author (S.J.M.) has substantially expanded the lateral nasal island flap to make it a reliable workhorse technique with specific indications and predictable aesthetic outcomes. We reviewed our data, which included 97 cases over 9 years to further define the lateral nasal island flap within the full range of nasal defects. An extended version of the lateral nasal island flap was used in 82 of the 97 cases. This lateral extended nasal island (LENI) myocutaneous flap is defined by the deep release of the flap off the piriform aperture and into the medial maxilla and is the main focus of this article.

## Methods

Institutional review board approval was obtained from the St Joseph Health Center for Clinical Research and informed written consent was waived owing to the deidentified data used in the study. Permission was obtained from the study patients whose photographs have been published. We performed a retrospective review of all patients who underwent the LENI flap procedure from 2009 to 2018 through a logged photo database (Google Sheets, 2018, Google Inc; and MediaPro, version 1.3.1.58487, Phase One Inc). Details on patient characteristics and indications for reconstruction are displayed in Table 1. We obtained data regarding the location and size of the defect, advancing distance of the described flap, and any additional procedures or stages. All patient medical records were reviewed for functional complications as a result of the flap. The 67 available postoperative photographs were reviewed to evaluate aesthetic outcome. The aesthetic outcome was rated on a scale from 1 to 3 by 2 independent observers. A score of 1 was given for almost unnoticeable reconstruction, a score of 2 for noticeable scar step-off or flap thickness, and a score of 3 for obvious deformity including tip and ala deformation. For all cases, the operative and postoperative notes were reviewed for surgical technique and complications. The numbers of procedure stages were defined by a flap or a scar revision. Minor procedures such as office dermabrasion, potassium titanyl phosphate laser treatment, or triamcinolone injection were not considered an additional stage. The dimensions of the defect and advancing distance of the flap were recorded. Bilateral LENI flaps were assumed to each advance approximately half of the defect dimension.

**Table 1.** Patient Characteristics for Lateral Extended Nasal Island Flap

Characteristic	Value
Sex, No. (%)	
Male	46 (56)
Female	36 (44)
Age, y	
Mean	64
Median (range)	73 (31-90)
Indication for surgery, No.	
Basal cell carcinoma	72
Squamous cell carcinoma	6
Squamous cell carcinoma in situ	1
Lentigo maligna	1
Postreconstructive deformity	2

The flaps included as lateral island flaps were angled at less than 45 degrees from the horizontal. At a 45 degree angle of inclination or higher, the flaps were classified as superolateral island flaps or superior nasal island flaps and were not included in the analysis.

**Figure 1.**
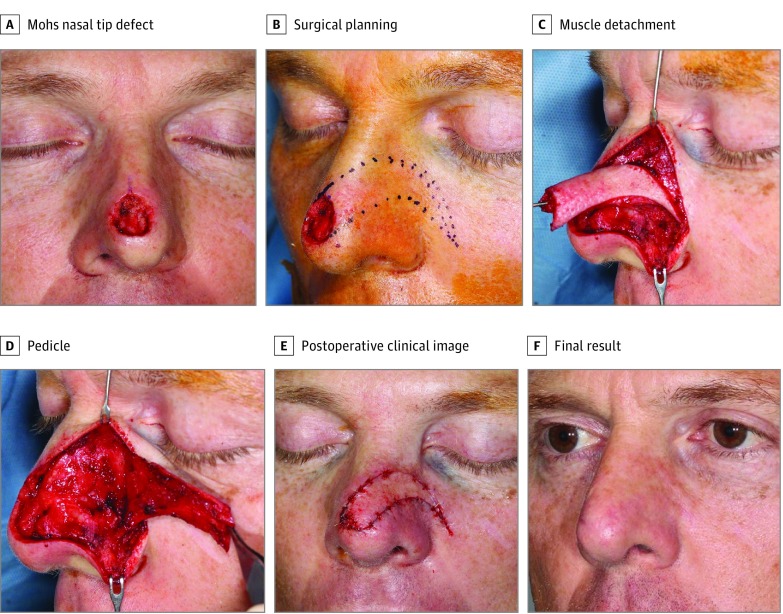
Construction of the Lateral Extended Nasal Myocutaneous Island Flap A, Final Mohs nasal tip defect size of 1.5 × 1.4 cm. B, Surgical planning. C, The muscle has been detached cephalically and caudally. D, Pedicle reflected laterally, showing its release from the piriform aperture and nasal bones. E, Immediate postoperative image. F, Final aesthetic result 11 weeks postoperatively.

The design of the flap extends laterally, following blood supply from the lateral nasal artery or branches of the alar artery.^8^ The flap incisions avoid extending caudal to the nasal alar groove which can be blunted if traversed. The incisions course away from the defect sides in parallel fashion (Figure 1). They converge and come to a point in an acute angle at a location designed by the surgeon. If the convergence point is chosen too medially, the donor site closing tension would retract the ala superiorly. If the convergence angle is made too obtuse, the donor site closure will result in a standing cone deformity. These are the design issues that extend the cutaneous pedicle into the cheek. The length of the lateral island flap is also based on the height of the defect. Because of convergence angle constraints, larger defects require the flap to extend into the medial cheek. So, the wider the defect, the longer the convergence of the flap sides.

The pedicle of the LENI flap distinguishes the flap from the simpler island or the simplest V-Y advancement flap. The lateral nasal muscles carry the blood supply from the lateral nasal and alar artery branches of the angular artery. The lateral nasal island flap is distinguished from the LENI flap by its deep flap release. The pedicle of the LENI flap extends past the nasal bones and the piriform aperture and is released from the bone. The skin island of the LENI flap can extend past nasal skin into the cheek but can also terminate more medially on the nose.

The angle of inclination of the flap advancement is determined by the location of the defect. Placement of incisions may be guided by anatomic subunit considerations such as avoidance of crossing the nasal alar groove. Adding a superior curve of the flap at the leading edge decreases the lateral to medial excursion demands of the flap by adding a superior to inferior advancement. The flap gains its length from the muscle pedicle elasticity, from the release of fascial attachments at the nasal bones and piriform aperture, the release of inelastic cheek skin from the cutaneous island, and by straightening the pedicle’s contour curve at the nasal cheek junction.

Once the flap direction is designed, the incisions are placed through the full thickness of the skin, leaving the underlying muscle fascia undisturbed. The dissection of the pedicle begins with slowly diverging subcutaneous elevation from the incisions over the muscle. The muscle pedicle is then elevated off the alar cartilages, vestibular skin if needed, and the nasal bones. If necessary, as in the case of the LENI flap, the deep fascia of the muscle pedicle is released from the piriform aperture and the medial maxilla. The muscle is cut superiorly and inferiorly to release it from its attachments thus narrowing the pedicle. The inferior (caudal) muscle release begins at the inferior cutaneous edge medially, parallels the skin edges for 1 cm, then diverges inferiorly to encompass sufficient blood supply in the muscle pedicle. The inferior muscle incision approaches the alar artery laterally. The artery can be spared and meticulously dissected to achieve greater release of the pedicle or cauterized and cut precisely. If additional release is required, the inferior and deep muscle release can further separate the muscle fascia from the alar base soft tissues at the most inferior piriform aperture curve.

The maximal release can allow the flap to advance up to 1.8 cm medially. To minimize distortion of the nasal tip or nasal valve collapse, cartilage grafting may be required for nasal tip strengthening. In some cases, partial defect closure is prudent to avoid nasal shape distortion. In those cases, additional flaps or skin grafts are required.

Secondary procedures may involve dermabrasion for sebaceous skin irregularity if needed. Some cases require a delayed sectioning of the muscle pedicle owing to its thickness. This thickness can impinge on the internal nasal valve impacting the airway. The secondary procedures are performed mostly under local anesthesia. Anticoagulation has not been found to be a contraindication to the primary procedure.

## Results

**Figure 2.**
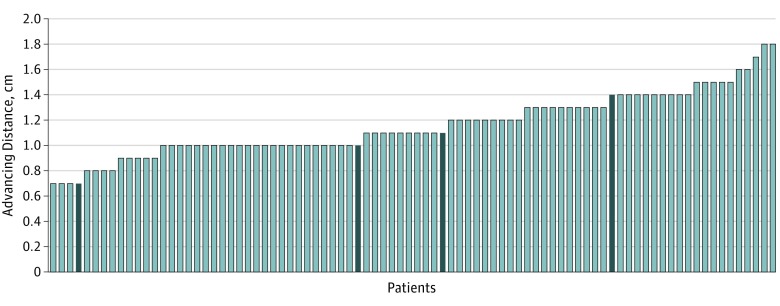
Histogram of Advancing Distances in the 86 Cases of the Lateral Extended Nasal Island (LENI) Flap in 82 Patients The dark bars indicate the 4 additional flaps that were included from 4 bilateral patients with LENI, resulting in a total of 86 LENI flaps in 82 patients.

Over the course of 9 years, 82 patients (46 men, 36 women) underwent nasal reconstruction with the lateral extended nasal island myocutaneous flap. Details on patient characteristics and indications for surgery are displayed in Table 1. Table 2 shows that the largest distribution of repairs was in the nasal tip with 52 cases (63%). The nasal dorsum had 21 cases (26%). Size analysis was defined by the distance of the flap advancement and not by the maximal dimension of the defect. In other words, our measurements defined mostly the width of the defect, not its height. The mean (SD) advancing distance was 1.2 (0.25) cm, whereas the mean for the maximal defect dimension was 1.3 cm. Maximal advancement distance was 1.8 cm (Figure 2). Of the 4 bilateral LENIs, the advancing distances of each flap were approximately half of the defect width. Bilateral flaps bridged the defect sizes of 1.4 cm, 2.0 cm, 2.2 cm, and 2.7 cm.

**Table 2.** Number of Flaps Organized by Advancing Distance, Location of Defect

Defect Location	Advancing Distance Ranges, cm			Total
	<1.0	1.0-1.5	1.5-2.0	
Nasal				
Tip	8	38	6	52
Dorsum	1	17	3	21
Sidewall	2	4	1	7
Soft tissue triangle	0	1	1	2
Total	11	60	11	82

**Figure 3.**
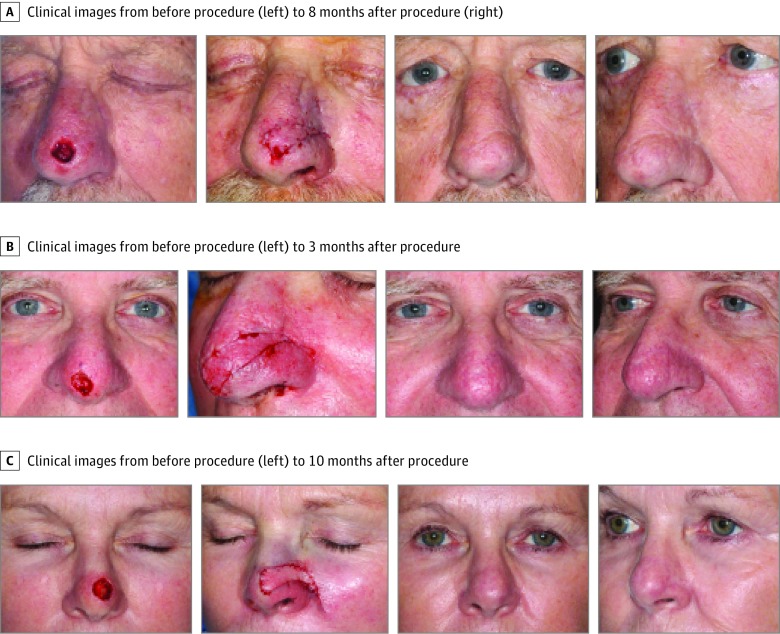
Before and After Photo Series of Lateral Extended Nasal Island (LENI) Myocutaneous Flap A, 1.5 × 1.3-cm defect size on nasal tip, 1.5-cm advancing distance and final aesthetic outcome 8 months postoperatively. B, 1.2 × 1-cm defect size on infratip, 1-cm advancing distance, and final aesthetic outcome 3 months postoperatively. C, 1.2 × 1.4-cm defect size on nasal tip, 1.4-cm advancing distance, and final aesthetic outcome 10 months postoperatively.

Of 82 patients, 67 had postoperative photographs available for aesthetic assessment (Figure 3). There were 44 patients (66%) with a rating score of 1 (undetectable or minimally noticeable reconstruction), 20 patients (30%) had a rating score of 2 (visible scar step-off or flap thickness), and 3 patients (4%) had a rating score of 3 (obvious deformity: twisted tip, nasal asymmetry). The LENI myocutaneous flap was used as a single-stage procedure in 65 cases (79%), a 2-stage procedure in 16 cases (20%), and a 3-stage procedure in 2 cases (2%).

The 15 simpler lateral nasal island flaps were employed for advancing distances of 0.5 cm to 1.2 cm (mean, 0.9 cm). Two-thirds of the lateral island flaps were performed in the first 3 years of the senior author’s (S.J.M.) experience. The author was clearly favoring the regular lateral nasal island flap for smaller defects and with early experience. Of the 82 patients with LENI, 3 had additional techniques for defect closure (skin grafts or other flaps). In addition, 4 patients each required bilateral LENI flaps to close the defect. No flap ischemia occurred. Nasal valve stenosis was a limited problem in a few flaps that resolved with steroid injections, time, or a second-stage debulking procedure.

## Discussion

Lateral nasal island flaps have been described generally in the decade between 1980 and 1990, but have not reached a widespread appeal. The limited popularity of the traditional flaps has been hampered by vague descriptions of technique, poorly defined indications, and limited applications.

In this study, we describe our modification of the lateral island flap, the LENI myocutaneous flap. The LENI flap’s versatility is its ability to routinely bridge 1.5-cm defects and its exceptional results as a 1-stage reconstruction in many cases. It is the deep release of the muscle fascia from piriform aperture and medial maxilla that defines the extended myocutaneous island flaps. That deep fascial detachment and the extensive superior and inferior muscle release achieves the flap’s mobility. The extension of the cutaneous island into the medial cheek is always associated with the LENI flaps in this study. The extended cutaneous island in our series eliminates the distortion of the ala compared with the shorter island flaps described in the 1980s.

The blood supply of the LENI flap is based on branches of the facial and angular arteries including alar artery and lateral nasal artery branches. Occasionally, alar artery division is required leaving the LENI flap supplied by the lateral nasal artery branches without compromising its vascularity. Its robust vascular supply affords raising the flap even in the setting of previous surgical scars. Despite the extensive release of flap attachments, its vascularity is reliably maintained.

The LENI myocutaneous flap was used primarily in the lower third of the nose in this study owing to the referral bias to the tertiary referral center setting and owing to the flap’s unique applicability to the caudal nose.

The LENI flap can be reliably used for extension distances of up to 1.5 cm. We have successfully advanced the LENI flap up to 1.8 cm. Higher tension of closure owing to greater advancement was associated with deformation of the tip and nasal valve collapse. In those cases additional support grafts are required for stabilization. This can be done with cartilage grafts in the lateral nasal wall, nasal valve, and caudal septum-tip complex.

Bilateral flaps were used for larger defects and had the advantage of limiting distortion of nasal shape owing to symmetrical forces on the nose. Although they can bridge larger defects, the senior author (S.J.M.) prefers other single techniques for reconstruction of larger defects.

Unlike cited literature, this study defined the distance of flap advancement and not the maximal size of the defect. Maximal defect size can overestimate the effectiveness of the flap compared with its advancement distance.

In the 18 patients (21%) who required a second or third surgical procedure, pedicle debulking, scar step-off correction, and alar rim adjustment were performed. In some of those cases, debulking of the pedicle was required to relieve narrowing the nasal valve. Nonsurgical procedures such as triamcinolone injections were used for nasal sidewall fullness. They also treated nasal valve impingement by the pedicle and surrounding deep scar tissue. Dermabrasion was effective in smoothing scar irregularity in sebaceous skin of the nose.

The LENI flap is a single-stage reconstruction in 80% of cases based on these data. In fact, it is a single-stage reconstruction with excellent aesthetic outcome in most cases. Approximately two-thirds of our reviewed flaps had an appearance rating of 1. Our appearance rating was based on the available photographs after the first stage. The average time of evaluation after the procedure was 2 months. It is likely that the appearance rating would have improved with a longer period of observation. Flaps with appearance rating of 2 (scar visibility and flap thickness) can be easily improved with a simple scar revision and flap debulking. Many of our 2-stage flaps were revised for that purpose, eliminating the visible signs of surgery.

This flap is characterized for its efficiency of skin use. Minimal skin is discarded with this technique. For patients with multiple skin cancers in their lifetime, skin sparing techniques are advantageous.

### Limitations

The LENI myocutaneous flap limitation is the medial advancing distance of 1.5 to 1.8 cm. It is also limited by the nasal alar groove. Extent of dissection considerably caudal to the groove can result in alar distortion, as evidenced by the patients in the study with poor outcomes. Distortion of the nasal tip owing to flap tension requires structural cartilage grafting. Finally, visible scarring owing to scar inversion is a function of the thickness of the flap. Vertical scar contracture from depth to surface can be addressed with beveling of each side of the closure or a later scar revision.

## Conclusions

The LENI myocutaneous flap is a reliable technique for reconstruction of the cutaneous defects of the nose with excellent aesthetic outcomes. We believe that it should serve as a workhorse alternative for reconstruction of nasal defects of up to 1.8 cm.
